# ANNOUNCEMENTS & RESOURCES

**Published:** 2019-05-13

## British Council for Prevention of Blindness Grant Programme

**Figure:**
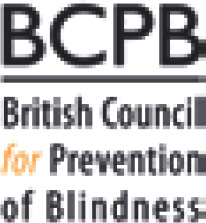


The British Council for Prevention of Blindness supports research into the prevention of blindness in low- and low-middle-income countries throughout the world. Grants are offered for research projects that further the goals of ‘VISION 2020: The Right to Sight’, in the following categories:

- Fellowships leading to the award of PhD or MD – up to £190,000 over 2 or 3 years
- Research grants – up to £60,000 (only awarded to UK universities and hospitals)
- Research Mentorship Awards – up to £15,000

We require applications to be sent both by email and as hard copy.

**Email closing date:** 27 September 2019 **Hard copy closing date:** 4 October 2019

For more details, full terms & conditions and application forms, please see **www.bcpb.org** or contact Diana Bramson, Charity Manager, BCPB, 4 Bloomsbury Square, London WC1A 2RP. Telephone: +44 20 7404 7114. Email: **info@bcpb.org**

BCPB is a registered charity – number 270941

## CEHJ app

Work is underway to develop an Android and iPhone app for the *Community Eye Health Journal*. The CEHJ app will make it possible to download articles and issues to read – and search – even when you are offline.

**Figure:**
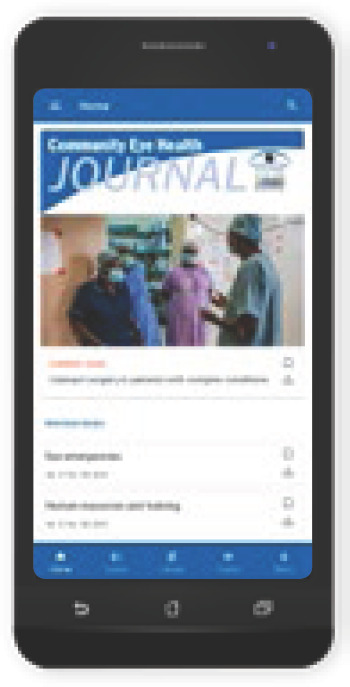


If you would like to be notified when the app becomes available, please subscribe to our email newsletter here: **www.cehjournal.org/subscribe/**

Your data is secure and will not be shared with others.

## Erratum

Due to a printing error, the last three bullet points in the article ‘Emergency management: acute endophthalmitis’ were not printed.

The instructions for preparing ceftazidime 2 mg/0.1 ml for intravitreal injection are as follows:

### Ceftazidime 2 mg/0.1 ml

- Reconstitute 500 mg vial with 10 ml saline
- Withdraw all 10 ml into 10 ml syringe
- Inject 2 ml of this solution back into vial
- Add 3 ml saline into vial to make up to 5 ml (20 mg/ml)
- Use 1 ml syringe to draw 0.1 ml of this solution (2 mg/0.1 ml)

The corrected article is available here:

**www.cehjournal.org/article/emergency-management-acute-endophthalmitis/**

## Courses

### MSc Public Health for Eye Care, London School of Hygiene & Tropical Medicine, London, UK

Fully funded scholarships are available for Commonwealth country nationals. For more information visit **www.lshtm.ac.uk/study/masters/mscphec.html** or email **romulo.fabunan@lsthm.ac.uk**

### Small Incision Cataract Surgery Training at Lions Medical Training Centre in Nairobi, Kenya

Courses begin every six weeks and cost US $1,000 for training and approximately US $1,000 for accommodation. Email **training@lionsloresho.org** or call/message +254 728 970 601 or +254 733 619 191.

#### Myopia calculator and courses

**Brien Holden Vision Institute Academy** offers a series of online courses (free and paid) and a free online myopia calculator that allows practitioners to model the impact of of various strategies to control the progression of myopia. Visit **https://academy.brienholdenvision.org**

## Subscriptions

Contact Anita Shah **admin@cehjournal.org**

### Subscribe to our mailing list

**web@cehjournal.org** or visit **www.cehjournal.org/subscribe**

### Visit us online

**www.cehjournal.org www.facebook.com/CEHJournal https://twitter.com/CEHJournal**

